# Pandemic Preparedness: The Importance of Adequate Immune Fitness

**DOI:** 10.3390/jcm11092442

**Published:** 2022-04-26

**Authors:** Pantea Kiani, Jessica Balikji, Aletta D. Kraneveld, Johan Garssen, Gillian Bruce, Joris C. Verster

**Affiliations:** 1 Division of Pharmacology, Utrecht Institute for Pharmaceutical Sciences, Utrecht University, 3584 CG Utrecht, The Netherlands; p.kiani@uu.nl (P.K.); j.balikji@uu.nl (J.B.); a.d.kraneveld@uu.nl (A.D.K.); j.garssen@uu.nl (J.G.); 2 Lead Healthcare, Luitenant Generaal van Heutszlaan 8, 3743 JN Baarn, The Netherlands; 3 Global Centre of Excellence Immunology, Nutricia Danone Research, 3584 CT Utrecht, The Netherlands; 4 Division of Psychology and Social Work, School of Education and Social Sciences, University of the West of Scotland, Paisley PA1 2BE, UK; gillian.bruce@uws.ac.uk; 5 Centre for Human Psychopharmacology, Swinburne University, Melbourne, VIC 3122, Australia

**Keywords:** pandemic preparedness, SARS-CoV-2, COVID-19, immune fitness, underlying disease, age, sex

## Abstract

Pandemic preparedness is an important issue in relation to future pandemics. The two studies described here aimed to identify factors predicting the presence and severity of coronavirus disease 2019 (COVID-19) symptoms. The CLOFIT study comprised an online survey among the Dutch population (*n* = 1415). Perceived immune fitness before the pandemic (2019) and during the first lockdown period (15 March–11 May 2020) and the number and severity of COVID-19 symptoms were assessed. The COTEST study, conducted between December 2020 and June 2021, replicated the CLOFIT study in *n* = 925 participants who were tested for severe acute respiratory syndrome coronavirus 2 (SARS-CoV-2) in Dutch commercial test locations. The CLOFIT study revealed that immune fitness before the pandemic was the greatest predictor of the number and severity of COVID-19 symptoms (20.1% and 19.8%, respectively). Other significant predictors included immune fitness during the lockdown (5.5% and 7.1%, respectively), and having underlying diseases (0.4% and 0.5%, respectively). In the COTEST study, for those who tested positive for SARS-CoV-2, immune fitness before the pandemic was the single predictor of the number (27.2%) and severity (33.1%) of COVID-19 symptoms during the pandemic. In conclusion, for those who tested positive for SARS-CoV-2, immune fitness before the pandemic was the strongest predictor of the number and severity of COVID-19 symptoms during the pandemic. Therefore, the development of strategies to maintain an adequate immune fitness must be regarded as an essential component of pandemic preparedness.

## 1. Introduction

The coronavirus disease 2019 (COVID-19) pandemic has had a significant negative impact on all aspects of society, including negative socioeconomic, psychological, and health consequences [1]. Perhaps the most important lesson learned from the COVID-19 pandemic is that adequate ‘pandemic preparedness’ is essential to combat future pandemics.

While a substantial number of people became infected with severe acute respiratory syndrome coronavirus 2 (SARS-CoV-2) during the first months of COVID-19 pandemic, the data also revealed that the chances of hospitalization and death were not equally distributed among the population [2]. Scientific literature revealed several vulnerable subpopulations who experienced more severe COVID-19 symptoms and had a higher risk of hospitalization due to COVID-19, including people of older age [3] and those having an underlying chronic disease such as obesity, diabetes, or cardiovascular disease [4,5]. Having a poorer immune fitness, often expressed as chronic systemic low-grade inflammation, is the commonality between these vulnerable groups [6,7].

Adequate immune fitness (i.e., a resilient immune system) refers to having the inbuilt capacity to adapt to external health challenges by establishing, maintaining, and regulating an appropriate immune response in order to prevent or resolve disease. Adequate immune fitness is thus essential to defend the body against exposure to external risks such as bacteria and viruses. While there are biomarkers to assess functioning of the immune system (e.g., cytokines), there is no biomarker that adequately corresponds to the overall concept of immune fitness. There are, however, scales developed to subjectively assess perceived immune fitness, such as the Immune Status Questionnaire, which captures immune fitness over a longer period of time, as well as a single item rating to assess perceived immune fitness real-time or retrospective at a specific time point [8,9].

A resilient society, i.e., of people who adopt a healthy lifestyle and therefore maintain an adequate immune fitness, has a better resistance to combat future pathogens including viruses. Poor immune fitness is caused in part by genetic predisposition, but it is also significantly impacted by lifestyle factors of the patient. For example, chronic intake of unhealthy food, lack of physical exercise, smoking, alcohol and drug use, chronic stress, and poor sleep all contribute to poorer levels of immune fitness [10]. It can therefore by hypothesized that chronic systemic low-grade inflammation is a risk factor for experiencing a greater number and more severe COVID-19 symptoms. Indeed, previous publications have suggested that an adequate immune fitness can improve the efficiency of innate and adaptive immune systems to either prevent or fight off COVID-19 [11,12].

The world’s strategy to prevent severe COVID-19 symptoms and hospitalization has predominantly been focused on earlier detection, vaccination against the SARS-CoV-2 virus, and to a lesser extent on the development of treatments for COVID-19. However, vaccines and medicines are not equally available around the world, leaving parts of the world (e.g., low-income countries) poorly protected against SARS-CoV-2 infection, or without optimal treatment once infected. Further, although for the COVID-19 pandemic vaccines were developed in unique record time, there is no guarantee that this will also be the case in future pandemics. Therefore, preparedness for future pandemics should not solely rely on the development of vaccines and medications, and studies should identify additional (low-cost and directly available) strategies that can easily be adopted by the general population. The aim of the two studies presented here was to investigate whether maintaining an adequate immune fitness would have a significant added value to the existing approaches of pandemic preparedness, as it significantly reduces the presence and severity of COVID-19 symptoms.

In the CLOFIT (Corona lockdown: how fit are you?) study, a retrospective survey was conducted concerning the first lockdown period in the Netherlands, from 15 March to 11 May 2020, during which the Alpha-variant of SARS-CoV-2 was present [11]. In the COTEST (Corona test street) study, the number and severity of COVID-19 symptoms was assessed in real-time among participants who were tested for SARS-CoV-2 infection in Dutch commercial test locations. The COTEST study was conducted between December 2020 and June 2021, when the Delta-variant of SARS-CoV-2 was present. For both studies it was hypothesized that for participants with a poorer immune fitness, the number and severity of COVID-19 symptoms would be significantly higher than for participants with an adequate immune fitness.

## 2. Methods

Both studies were approved by the Ethics Committee of the Faculty of Social and Behavioral Sciences of Utrecht University (approval code: FETC17-061), and electronic informed consent was obtained from all participants.

The CLOFIT study comprised an anonymous online survey via SurveyMonkey, which was conducted between 24 June and 26 July 2020 [13]. Participants were recruited via Facebook advertisements distributed among the Dutch adult population (>18 years old). In addition to recording demographics, participants retrospectively reported on: their past year’s perceived immune fitness, referred to as ‘immune fitness (2019)’; their perceived immune fitness during the first lockdown period in the Netherlands (15 March–11 May 2020), referred to as ‘immune fitness (DL)’; and the presence and severity of symptoms associated with COVID-19 during the lockdown. A detailed description of the study methodology of the CLOFIT study is published elsewhere [13].

In the COTEST study, Dutch adults (18 years and older) who conducted a SARS-CoV-2 rapid antigen test between December 2020 and June 2021 at one of the Lead Healthcare test locations across the Netherlands were invited to participate in the study. Participants received the outcome of the SARS-CoV-2 test via email. The email contained an invitation to complete an online survey on COVID-19 via SurveyMonkey.

The surveys comprised assessments of demographic data, including age, sex, body weight, and height to compute the Body Mass Index (BMI). Underlying diseases, i.e., chronic health conditions, as listed by the National Institute for Public Health and the Environment (RIVM), were assessed [14]. These included cardiovascular disease or hypertension, diabetes, liver disease, neurological diseases (e.g., epilepsy, migraine), immune disorders (e.g., rheumatism, Crohn disease), allergy (e.g., hay fever), kidney disease, pulmonary diseases (e.g., chronic obstructive pulmonary disease, asthma), anxiety, depression, sleep disorders, and “other” to report any unlisted disease. Immune fitness (2019) was assessed with the Immune Status Questionnaire (ISQ) [8]. The 7-item ISQ included ‘common cold’, ‘diarrhea’, ‘sudden high fever’, ‘headache’, ‘muscle and joint pain’, ‘skin problems (e.g., acne & eczema)’, and ‘coughing’. Participants indicated how often they experienced these immune-related complaints in 2019. They could choose among the answering possibilities ‘never’, ‘sometimes’, ‘regularly’, ‘often’, and ‘(almost) always’. The overall ISQ score, after recoding [10], ranges from 0 (poor) to 10 (excellent). Higher ISQ scores correspond to a better perceived immune status. Immune fitness (DL) was assessed using a 1-item scale ranging from 0 (poor) to 10 (excellent) [8,9]. In the CLOFIT study, nine symptoms associated with the Alpha variant of SARS-CoV-2, as listed by the RIVM, were assessed [13,15]. These nine symptoms included sneezing, running nose, sore throat, coughing, malaise/feeling sick, high temperature (up to 38 Celsius), fever (38 Celsius and higher), shortness of breath, and chest pain. In the COTEST study, 17 symptoms listed by the US Centers for Disease Control for the Delta-variant of SARS-CoV-2 were assessed [16]. The 17 symptoms included running nose, sore throat, coughing, fever (38 Celsius and higher), shortness of breath, chest pain, congestion, headache, shivering, chest pain, fatigue, muscle pain, loss of smell or taste, confusion, difficulty waking up/staying awake, blueish lips or face, nausea or vomiting, and diarrhea. The average severity score and number of symptoms was computed. Perceived immune fitness was assessed at the time of testing (referred to as ‘immune fitness (T)’) using a 1-item scale ranging from 0 (poor) to 10 (excellent) [8,9]. In the COTEST study, all participants were tested for SARS-CoV-2, at one of the Lead Healthcare test street facilities across the Netherlands, using the Panbio^TM^ COVID-19 antigen rapid test device (Abbott Diagnostic GmbH, Jena, Germany) or the Roche SARS-CoV-2 rapid antigen test (Roche Diagnostics, Basel, Switzerland). Samples were analyzed by trained personnel of Lead Healthcare. Within 30 min, participants received an email with the outcome of the test. The outcome could be positive (SARS-CoV-2 detected) or negative (no SARS-CoV-2 infection).

The data were analyzed with SPSS (IBM Corp. Released 2013. IBM SPSS Statistics for Windows, Version 28.0. Armonk, NY, USA: IBM Corp.). Differences between groups were compared using the Independent Samples Mann–Whitney U Test or Chi-squared test and considered significant if *p* < 0.05. Spearman’s correlations were computed to evaluate relationships between the variables. Correlations were considered significant if *p* < 0.05. Stepwise linear regression analyses were conducted to identify variables that significantly predict the number or severity of symptoms associated with COVID-19. Variables included in the analyses were age, sex, BMI, immune fitness (2019), and immune fitness (DL) or immune fitness (T).

## 3. Results

### 3.1. CLOFIT Study

Data from *n* = 1415 participants were evaluated. Their ages ranged from 18 to 94 years old. As this study was conducted at the beginning of the COVID-19 pandemic, 95% of the sample was not tested for SARS-CoV-2. Their demographics and study outcomes are summarized in Table 1.

Women were significantly younger and had a lower BMI than men (see Table 1). Both immune fitness (2019) (*p* < 0.001) and immune fitness (DL) (*p* < 0.001) were significantly poorer in women than in men. Women also reported experiencing a significantly greater number and more severe COVID-19 symptoms than men. Weak correlations were found between age and the number (r = −0.079, *p* = 0.011) and severity (r = −0.063, *p* = 0.043) of COVID-19 symptoms. No significant correlations were found between BMI and the number (r = −0.008, *p* = 0.791) and severity (r = −0.003, *p* = 0.929) of COVID-19 symptoms. Of the sample, N = 920 (65.5%) reported having one or more underlying chronic disease. Of them, more than half of the participants reported having one underlying disease (*n* = 518, 56.3%) and the other participants reported a combination of two or more underlying chronic diseases.

Significant correlations were found between immune fitness (2019) and the number (r = −0.455, *p* < 0.001) and severity of COVID-19 symptoms (r = −0.461, *p* < 0.001). Immune fitness (DL) also correlated significantly with the number (r = −0.348, *p* < 0.001) and severity of COVID-19 symptoms (r = −0.367, *p* < 0.001).

A stepwise regression analysis, including the predictor variables sex, age, BMI, underlying disease, immune fitness (2019), and immune fitness (DL), revealed a significant model explaining 26.0% of variance in the number of reported COVID-19 symptoms. The three variables that were significant predictors of the number of COVID-19 symptoms were immune fitness (2019) (20.1%), immune fitness (DL) (5.5%), and having an underlying disease (0.4%). A second analysis revealed a significant model explaining 27.4% of variance in the severity of COVID-19 symptoms. The three variables that were significant predictors of the severity of COVID-19 symptoms were immune fitness (2019) (19.8%), immune fitness (DL) (7.1%), and having an underlying disease (0.5%).

### 3.2. COTEST Study

A total of *n* = 925 Dutch adults participated in the study. A total of *n* = 88 tested positive and *n* = 837 tested negative for SARS-CoV-2. Their demographics and study outcomes are summarized in Table 2. No significant differences were found between the groups.

On average, those who tested positive for SARS-CoV-2 reported a mean (SD) of 5.2 (3.2) symptoms, which was significantly greater than the mean (SD) number of symptoms reported by those who tested negative for SARS-CoV-2 (mean: 3.4, SD = 3.0, *p* < 0.001). Of the group that tested positive, 8.0% of participants (7 out of 88) reported no COVID-19 symptoms, whereas of the group that tested negative, 20.9 % of participants (175 out of 837) reported no COVID-19 symptoms. This difference in percentage of asymptomatic participants was statistically significant (*p* = 0.004).

**Figure 1:**
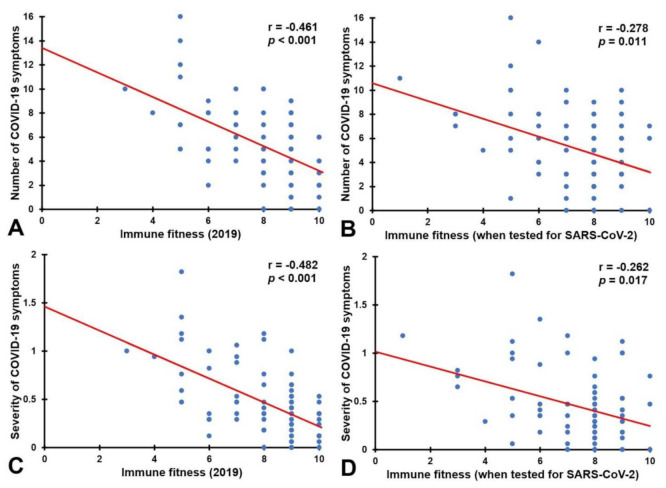
Relationship between immune fitness and the number and severity of COVID-19 symptoms for participants that tested positive for SARS-CoV-2. Shown is the relationship between the number of COVID-19 symptoms and immune fitness (2019) (**A**) and immune when tested for SARS-CoV-2 (**B**), and the relationship between the severity of COVID-19 symptoms and immune fitness (2019) (**C**) and immune fitness when tested for SARS-CoV-2 (**D**). The red lines represent the Spearman’s correlations. Correlations are considered significant if *p* < 0.05. Abbreviations: COVID-19 = coronavirus disease 2019; SARS-CoV-2 = severe acute respiratory syndrome coronavirus 2.

Table 3 shows that significant correlations were found between immune fitness (2019 and T) and the number and severity of symptoms associated with COVID-19. The correlations were significant for both participants who tested negative and for participants who tested positive for SARS-CoV-2. Whereas the correlations of immune fitness (2019) did not significantly differ in magnitude between the two groups, the correlations with immune fitness (T) were significantly more robust among those who tested negative (see Table 3). Figure 1 shows the correlations for participants that tested positive for SARS-CoV-2.

A stepwise regression analysis was conducted including the predictor variables sex, age, BMI, underlying disease, immune fitness (2019), and immune fitness (T). For those who tested negative for SARS-CoV-2, a significant model was found explaining 30.9% of variance in the number of reported COVID-19 symptoms. The three variables that were significant predictors of the number of COVID-19 symptoms were immune fitness (T) (20.0%), immune fitness (2019) (10.5%), and sex (0.4%). A second significant model explained 29.9% of variance in the severity of COVID-19 symptoms. The three variables that were significant predictors of the severity COVID-19 symptoms were immune fitness (T) (19.4%), immune fitness (2019) (10.0%), and sex (0.5%). For participants who tested positive for SARS-CoV-2, the analysis revealed that the models to predict the number and severity of COVID-19 symptoms had only one significant predictor variable: immune fitness (2019). Immune fitness (2019) predicted 27.2% of the number of COVID-19 symptoms, and 33.1% of the severity of COVID-19 symptoms.

## 4. Discussion

The data described here show that adequate immune fitness is associated with reporting a significantly lower number and severity of COVID-19 symptoms. Often, pandemic preparedness concerns the early and better detection of viruses, and the development of vaccines and medicines. Results from the CLOFIT and COTEST studies add that measures to maintain an adequate immune fitness should also be an essential component of pandemic preparedness.

The CLOFIT study revealed that immune fitness (2019) was the most important predictor of the number and severity of COVID-19 symptoms, followed by immune fitness (DL). Other potential predictors such as BMI, age, and sex did not significantly contribute to the model, and the impact of underlying diseases was marginal (<1.0%).

The COTEST study confirmed the strong association between immune fitness and the number and severity of COVID-19 symptoms. Although for the sample as a whole perceived immune fitness (T) was a stronger predictor than immune fitness (2019), for those that tested positive for SARS-CoV-2 immune fitness (2019) was the single predictor of the number and severity of COVID-19 symptoms. Of note, correlations between immune fitness (2019) and the number and severity of COVID-19 symptom scores were significant irrespective of whether participants tested positive or negative for SARS-CoV-2, confirming that the ISQ is a good predictor of future immune fitness per se.

It is important to keep in mind that the CLOFIT study covered the first COVID-19 lockdown in the Netherlands. None of the participants were vaccinated and testing opportunities for SARS-CoV-2 were still minimal (95% of the sample was not tested). Therefore, for most participants in the CLOFIT study it remains unknown whether they were infected with SARS-CoV-2 during the first lockdown. The symptoms assessed are common symptoms that can also be present when testing negative for SARS-CoV-2 (e.g., when having a common cold). It was therefore critical that the COTEST study was conducted in participants who were all tested for SARS-CoV-2 infection. The significant relationship of immune fitness (2019) with the number and severity of COVID-19 symptoms was evident in both studies.

The results of the presented studies suggest that measures to maintain an adequate immune fitness are vital to reducing the presence and severity of COVID-19 symptoms. Previous research also suggested that participants with chronic systemic low-grade inflammation, such as those with underlying chronic diseases, were more vulnerable to infection with SARS-CoV-2, reported greater COVID-19 symptom severity, and higher hospitalization rates [17,18]. Especially for these vulnerable groups it is critical to adopt a healthy lifestyle to maintain an adequate immune fitness and make themselves more resilient for future pandemics. However, maintaining a resilient immune system also plays an important role in pandemic preparedness for the relative healthy general population. Even without being hospitalized, being infected with SARS-CoV-2 can make people sick and require self-quarantine, which can have significant negative socioeconomic consequences and a negative impact on quality of life. Therefore, the general population will also significantly benefit from maintaining an adequate immune fitness. This can be achieved, at least in part, by adopting a healthy lifestyle. COVID-19 literature reveals that in addition to physical and mental health [19], adopting healthy lifestyle choices had a significant positive impact on immune fitness during the pandemic, including maintaining a healthy diet [20], regular physical activity [21], adequate sleep [22], a supportive environment [23], moderate alcohol consumption [24], and refraining from substance abuse [25]. In relation to pandemic preparedness, it is therefore essential that governments invest in improving the immune fitness of the general population and promote a healthy lifestyle. In addition, a healthy lifestyle and adequate immune fitness will also help to reduce the chances of developing non-communicable diseases [26].

There are some strengths and limitations that should be considered when interpreting the presented data. Firstly, in the CLOFIT study data were collected retrospectively. As such, recall bias may have affected accuracy. Therefore, in the COTEST study this data was collected shortly after being tested for SARS-CoV-2. Secondly, although the sample sizes of both studies are sufficiently large, the sample that tested positive for SARS-CoV-2 in the COTEST study (*n* = 88) was relatively small. The percentage of those who tested positive for SARS-CoV-2 (9.5%) does reflect the percentages reported for the Netherlands during the study period [27]. Thirdly, the CLOFIT sample was recruited via Facebook and the COTEST sample among those who conducted a SARS-CoV-2 test in Dutch commercial test streets. This may have caused selection bias, and therefore both study samples are not national representative samples. However, this was not a provision for the conducted analyses. Fourthly, the assessed COVID-19 symptoms differ between the CLOFIT and COTEST studies. This is related to the different periods when the studies were conducted, including different SARS-CoV-2 variants, and advancing scientific knowledge on COVID-19 symptomatology. That is, during the CLOFIT study the alpha variant of SARS-CoV-2 was dominant, whereas during the COTEST study the delta variant of SARS-CoV-2 was dominant. The assessed symptoms reflected those corresponding to these variants. Finally, the SARS-CoV-2 tests used in the COTEST study were rapid antigen tests. Given that polymerase chain reaction (PCR) tests are more accurate than antigen tests, there is a small chance of false positives among those allocated to the group of participants that tested positive for SARS-CoV-2. A Canadian study among 903,408 participants revealed a false positive rate of 0.05% for rapid antigen tests [28]. Extrapolating this percentage to the current study would suggest less than one false positive participant. Given this, we are confident that the study outcomes are accurate.

## 5. Conclusions

The preparedness for future pandemics should not solely rely on the development of vaccines and medications, but also focus on additional (low-cost and directly available) strategies that can easily be adopted by the general population. The current data support that maintaining an adequate immune fitness can make an essential contribution to pandemic preparedness. It is a cost-effective and easy to implement strategy to reduce the impact of future pandemics. Preventive campaigns should therefore inform the general public of the importance of adopting a healthy lifestyle and create awareness that maintaining an adequate immune fitness is important to defend themselves against experiencing severe disease symptoms during future pandemics.

## Figures and Tables

**Table 1:** Demographics and study outcomes of the CLOFIT study.

Variable	Overall	Men	Women	*p*-Value
*n* (%)	1415 (100%)	503 (35.5%)	912 (64.5%)	<0.001 *
Age (year)	45.0 (18.5)	49.7 (18.4)	42.4 (18.0)	<0.001 *
Height (m)	1.73 (0.09)	1.80 (0.08)	1.69 (0.07)	<0.001 *
Weight (kg)	79.3 (18.8)	87.6 (17.4)	74.8 (17.9)	<0.001 *
BMI (kg/m^2^)	26.4 (5.8)	26.9 (5.3)	26.2 (6.1)	<0.001 *
Underlying disease (% yes)	65.5	60.4	68.3	0.003 *
Immune fitness (2019)	7.1 (2.4)	7.8 (2.3)	6.8 (2.5)	<0.001 *
Immune fitness (DL)	7.1 (2.0)	7.4 (1.8)	6.9 (2.1)	<0.001 *
Number of COVID-19 Symptoms	2.7 (2.2)	2.4 (2.1)	2.8 (2.3)	0.010 *
Severity of COVID-19 Symptoms	0.44 (0.5)	0.38 (0.4)	0.48 (0.5)	0.001 *

Mean and standard deviation (SD, between brackets) are shown. Significant differences between men and women (*p* < 0.05) are indicated by *. Abbreviations: BMI = body mass index; COVID-19: coronavirus disease 2019, DL = during the first lockdown period.

**Table 2:** Demographics and study outcomes of the COTEST study.

Variable	Tested Positive	Tested Negative	*p*-Value
*n*	88	837	<0.001 *
Male/female (%)	60.2/39.8	54.5/45.5	0.313
Age (year)	46.3 (13.3)	47.0 (14.5)	0.697
BMI (kg/m^2^)	26.1 (4.4)	26.0 (4.3)	0.848
Underlying diseases (% yes)	58.0	54.1	0.502
Immune fitness (2019)	8.1 (1.7)	7.8 (2.0)	0.466
Immune fitness (T)	7.3 (1.7)	7.5 (1.6)	0.714
Number of COVID-19 symptoms	5.2 (3.2)	3.4 (3.0)	<0.001 *
Severity of COVID-19 symptoms	0.46 (0.4)	0.29 (0.3)	<0.001 *

Significant differences between those who tested positive or negative for SARS-CoV-2 infection (*p* < 0.05) are indicated by *. Abbreviations: BMI = body mass index; COVID-19: coronavirus disease 2019, T = assessed when tested for SARS-CoV-2.

**Table 3:** Relationship of immune fitness and the number and severity of COVID-19 symptoms.

Correlations with Immune Fitness (2019)							
Correlation with COVID-19 Symptoms	Overall		Tested Positive		Tested Negative		Comparison
	r	*p*-Value	r	*p*-Value	r	*p*-Value	*p*-Value
Number of symptoms	−0.431	<0.001 *	−0.461	<0.001 *	−0.442	<0.001 *	0.834
Severity of symptoms	−0.432	<0.001 *	−0.482	<0.001 *	−0.440	<0.001 *	0.638
**Correlations with immune fitness (T)**							
Number of symptoms	−0.451	<0.001 *	−0.278	0.011 *	−0.473	<0.001 *	0.044 *
Severity of symptoms	−0.459	<0.001 *	−0.262	0.017 *	−0.481	<0.001 *	0.024 *

Significant correlations (*p* < 0.05) are indicated by *. Abbreviations: COVID-19: coronavirus disease 2019, T = assessed when tested for SARS-CoV-2.

## Data Availability

The data are available upon reasonable request from the corresponding author.

## References

[1] Prati G., Mancini A.D. (2021). The psychological impact of COVID-19 pandemic lockdowns: A review and meta-analysis of longitudinal studies and natural experiments. Psychol. Med..

[2] National Institute for Public Health and the Environment (RIVM) Risicogroepen en COVID-19. https://www.rivm.nl/coronavirus-covid-19/risicogroepen.

[3] Lee K., Jeong G.-C., Yim J. (2020). Consideration of the psychological and mental health of the elderly during COVID-19: A theoretical review. Int. J. Environ. Res. Public Health.

[4] Mohammad S., Aziz R., Al Mahri S., Malik S.S., Haji E., Husain Khan A., Saleem Khatlani T., Bouchama A. (2021). Obesity and COVID-19: What makes obese host so vulnerable?. Immun. Ageing.

[5] Almeida Pititto B., Dualib P.M., Zajdenverg L., Rodrigues Dantas J., Dias de Souza F., Rodacki M., Casaccia Bertoluci M. (2020). Severity and mortality of COVID 19 in patients with diabetes, hypertension and cardiovascular disease: A meta-analysis. Diabetol. Metab. Syndr..

[6] Furman D., Campisi J., Verdin E., Carrera-Bastos P., Targ S., Franceschi C., Ferrucci L., Gilroy D.W., Fasano A., Miller G.W. (2019). Chronic inflammation in the etiology of disease across the life span. Nat. Med..

[7] Weyand C.M., Goronzy J.J. (2016). Aging of the immune system. Mechanisms and therapeutic targets. Ann. Am. Thorac. Soc..

[8] Wilod Versprille L.J.F., van de Loo A.J.A.E., Mackus M., Arnoldy L., Sulzer T.A.L., Vermeulen S.A., Abdulahad S., Huls H., Baars T., Kraneveld A.D. (2019). Development and validation of the Immune Status Questionnaire (ISQ). Int. J. Environ. Res. Public Health.

[9] Van Schrojenstein Lantman M., Otten L.S., Mackus M., de Kruijff D., van de Loo A.J.A.E., Kraneveld A.D., Garssen J., Verster J.C. (2017). Mental resilience, perceived immune functioning, and health. J. Multidiscip. Healthc..

[10] Vrousgos G. (2016). Lifestyle Factors that can induce an independent and persistent low-grade systemic inflammatory response: A wholistic approach. Open Med. J..

[11] Taghizadeh-Hesary F., Akbari H. (2020). The powerful immune system against powerful COVID-19: A hypothesis. Med. Hypotheses.

[12] Sette A., Crotty S. (2021). Adaptive immunity to SARS-CoV-2 and COVID-19. Cell.

[13] Kiani P., Merlo A., Saeed H.M., Benson S., Bruce G., Hoorn R., Kraneveld A.D., Severeijns N.R., Sips A.S.M., Scholey A. (2021). Immune fitness, and the psychosocial and health consequences of the COVID-19 pandemic lockdown in The Netherlands: Methodology and design of the CLOFIT study. Eur. J. Investig. Health Psychol. Educ..

[14] National Institute for Public Health and the Environment (RIVM) Aandoeningen. Welke Aandoeningen Hebben We in De Toekomst?. https://www.vtv2018.nl/aandoeningen.

[15] RIVM Symptoms. https://www.rivm.nl/coronavirus-covid-19/ziekte.

[16] Centers for Disease Control and Prevention Symptoms of COVID-19. https://www.cdc.gov/coronavirus/2019-ncov/symptoms-testing/symptoms.html.

[17] Hulme K.D., Noye E.C., Short K.R., Labzin L.I. (2021). Dysregulated inflammation during obesity: Driving disease severity in influenza virus and SARS-CoV-2 infections. Front. Immunol..

[18] Kompaniyets L., Pennington A.F., Goodman A.B., Rosenblum H.G., Belay B., Ko J.Y., Chevinsky J.R., Schieber L.Z., Summers A.D., Lavery A.M. (2021). Underlying medical conditions and severe illness among 540,667 adults hospitalized with COVID-19, March 2020–March 2021. Prev. Chronic. Dis..

[19] Liu L., Ni S.Y., Yan W., Lu Q.D., Zhao Y.M., Xu Y.Y., Mei H., Shi L., Yuan K., Han Y. (2021). Mental and neurological disorders and risk of COVID-19 susceptibility, illness severity and mortality: A systematic review, meta-analysis and call for action. EClinical Med..

[20] Vishwakarma S., Panigrahi C., Barua S., Sahoo M., Mandliya S. (2022). Food nutrients as inherent sources of immunomodulation during COVID-19 pandemic. Lebensm. Wiss. Technol..

[21] Filgueira T.O., Castoldi A., Santos L., de Amorim G.J., de Sousa Fernandes M.S., Anastácio W., Campos E.Z., Santos T.M., Souto F.O. (2021). The relevance of a physical active lifestyle and physical fitness on immune defense: Mitigating disease burden, with focus on COVID-19 consequences. Front. Immunol..

[22] Schmitz N., van der Werf Y.D., Lammers-van der Holst H.M. (2022). The importance of sleep and circadian rhythms for vaccination success and susceptibility to viral infections. Clocks Sleep.

[23] Hendriksen P.A., Kiani P., Garssen J., Bruce G., Verster J.C. (2021). Living alone or together during lockdown: Association with mood, immune fitness and experiencing COVID-19 symptoms. Psychol. Res. Behav. Manag..

[24] Merlo A., Severeijns N.R., Benson S., Scholey A., Garssen J., Bruce G., Verster J.C. (2021). Mood and changes in alcohol consumption in young adults during COVID-19 lockdown: A model explaining associations with perceived immune fitness and experiencing COVID-19 symptoms. Int. J. Environ. Res. Public Health.

[25] Wang Q.Q., Kaelber D.C., Xu R., Volkow N.D. (2021). COVID-19 risk and outcomes in patients with substance use disorders: Analyses from electronic health records in the United States. Mol. Psychiat..

[26] Nyberg S.T., Singh-Manoux A., Pentti J., Madsen I., Sabia S., Alfredsson L., Bjorner J.B., Borritz M., Burr H., Goldberg M. (2020). Association of healthy lifestyle with years lived without major chronic diseases. JAMA Int. Med..

[27] National Institute for Public Health and the Environment (RIVM) Weekcijfers Coronavirus SARS-CoV-2. https://www.rivm.nl/coronavirus-covid-19/weekcijfers.

[28] Gans J.S., Goldfarb A., Agrawal A.K., Sennik S., Stein J., Rosella L. (2022). False-Positive Results in Rapid Antigen Tests for SARS-CoV-2. JAMA.

